# Supplementary material for: Cell cycle constraints on capsulation and bacteriophage susceptibility
Source: eLife. 2014 Nov 25;3:e03587. doi: 10.7554/eLife.03587 (PMC4241560; doi:10.7554/eLife.03587)
Supplement: Supplementary file 1. — Phenotypes of WT Caulobacter and mutant strains. DOI: http://dx.doi.org/10.7554/eLife.03587.029 [file elife03587s002.docx]

**Table S1. Phenotypes of *WT* *Caulobacter* and mutant strains.**

Mutants were complemented with the corresponding gene on a medium-copy number plasmid (pMT335) under control of P*_van_*. For cell density the presence of high buoyancy (L) or low buoyancy (H) band is indicated. Mucoidy was verified on PYE agar plates containing 3% sucrose. Sensitivity to φCr30 was assessed by spot tests on lawns of cells.

| **Strain** | **Cell density** | **Mucoidy** | **Sensitivity to φCr30** |
| --- | --- | --- | --- |
| ***WT* pMT335** | L/H | + | + |
| ***WT* pMT335-*hvyA*** | H | - | + |
| ***WT* pSA362** | L/H | + | + |
| ***WT* pSA361** | L/H | + | + |
| ***WT* pSA401** | L/H | + | + |
| ***WT* pSA62** | L/H | + | + |
| ***WT* pSA324** | L/H | + | + |
| ***WT* pUG35** | L/H | + | + |
| ***WT* pSA102** | L/H | + | + |
| **Δ*pleC* pMT335** | L | + | - |
| **Δ*pleC* pMT335-*hvyA*** | H | - | + |
| **Δ*pleC* pSA362** | L | + | - |
| **Δ*pleC* pSA361** | L | + | - |
| **Δ*pleC* pSA401** | L | + | - |
| **Δ*pleC* pSA62** | L | + | - |
| **Δ*pleC* pSA324** | L | + | - |
| **Δ*pleC* pUG35** | L | + | - |
| **Δ*pleC* pSA102** | L | + | - |
| **Δ*hvyA* pMT335** | L | + | - |
| **Δ*hvyA* pMT335-*hvyA*** | H | - | + |
| **Δ*pleC* Δ*hvyA* pMT335** | L | + | - |
| **Δ*pleC* Δ*hvyA* pMT335-*hvyA*** | H | - | + |
| **Δ*CCNA_00162* pMT335** | H | - | + |
| **Δ*CCNA_00162* pSA362** | L/H | + | + |
| **Δ*pleC* Δ*CCNA_00162* pMT335** | H | - | + |
| **Δ*pleC* Δ*CCNA_00162* pSA362** | L | + | - |
| **Δ*CCNA_00163* pMT335** | H | - | + |
| **Δ*CCNA_00163* pSA361** | L/H | + | + |
| **Δ*pleC* Δ*CCNA_00163* pMT335** | H | - | + |
| **Δ*pleC* Δ*CCNA_00163* pSA361** | L | + | - |
| **Δ*CCNA_00164* pMT335** | H | - | + |
| **Δ*CCNA_00164* pSA401** | L/H | + | + |
| ***pleC*::Tn5 Δ*CCNA_00164* pMT335** | H | - | + |
| ***pleC*::Tn5 Δ*CCNA_00164* pSA401** | L | + | - |
| **Δ*CCNA_00167* pMT335** | H | - | + |
| **Δ*CCNA_00167* pSA62** | L/H | + | + |
| **Δ*pleC* Δ*CCNA_00167* pMT335** | H | - | + |
| **Δ*pleC* Δ*CCNA_00167* pSA62** | L | + | - |
| ***CCNA_00168*::Tn pMT335** | H | - | + |
| ***CCNA_00168*::Tn pSA324** | L/H | + | + |
| **Δ*pleC* *CCNA_00168*::Tn pMT335** | H | - | + |
| **Δ*pleC* *CCNA_00168*::Tn pSA324** | L | + | - |
| **Δ*CCNA_03998* pMT335** | H | - | + |
| **Δ*CCNA_03998* pUG35** | L/H | + | + |
| **Δ*pleC* Δ*CCNA_03998* pMT335** | H | - | + |
| **Δ*pleC* Δ*CCNA_03998* pUG35** | L | + | - |
| **Δ*CCNA_00470* pMT335** | H | - | + |
| **Δ*CCNA_00470* pSA102** | L/H | + | + |
| **Δ*pleC* Δ*CCNA_00470* pMT335** | H | - | + |
| **Δ*pleC* Δ*CCNA_00470* pSA102** | L | + | - |
| **Δ*hvyA* Δ*CCNA_00163*** | H | - | + |
| **Δ*hvyA* Δ*CCNA_00167*** | H | - | + |
| **Δ*hvyA* Δ*CCNA_00470*** | H | - | + |
| **Δ*pleC* Δ*hvyA* Δ*CCNA_00167*** | H | - | + |
| **Δ*pssY*** | L/H | + | + |
| **Δ*pleC* Δ*pssY*** | L | + | - |
