# Supplementary material for: Cell cycle constraints on capsulation and bacteriophage susceptibility
Source: eLife. 2014 Nov 25;3:e03587. doi: 10.7554/eLife.03587 (PMC4241560; doi:10.7554/eLife.03587)
Supplement: Supplementary file 2. — Nucleotide position of himar1 insertions. DOI: http://dx.doi.org/10.7554/eLife.03587.030 [file elife03587s003.docx]

**Table S2. Nucleotide position of *himar1* insertions.**

(*C. crescentus* NA1000 chromosome, accession NC_011916)

| **Insertions in Δ*pleC*** | |
| --- | --- |
| Nucleotide position | Gene |
| 172151 | *CCNA_00162* |
| 172788 | *CCNA_00162* |
| 172827 | *CCNA_00162* |
| 175141 | *CCNA_00163* |
| 175361 | *CCNA_00164* |
| 179520 | *CCNA_00167* |
| 179712 | *CCNA_00167* |
| 180672 | *CCNA_00168* |
| 475658 | *CCNA_03998* |
| 475699 | *CCNA_03998* |
| 475701 | *CCNA_03998* |
| 475787 | *CCNA_03998* |
| 475788 | *CCNA_03998* |
| 475792 | *CCNA_03998* |
| 475925 | *CCNA_03998* |
| 476186 | *CCNA_03998* |
| 485145 | *CCNA_00470* |
| 485260 | *CCNA_00470* |
| 485525 | *CCNA_00470* |
| 485592 | *CCNA_00470* |
| 485695 | *CCNA_00470* |
| 485698 | *CCNA_00470* |
| 486183 | *CCNA_00471* |
| 486767 | *CCNA_00471* |
| 486858 | *CCNA_00471* |
| **Insertions in Δ*hvyA*** | |
| 174524 | *CCNA_00163* |
| 180563 | *CCNA_00168* |
| 475601 | *CCNA_03998* |
| 476034 | *CCNA_03998* |
| 476188 | *CCNA_03998* |
| 485259 | *CCNA_00470* |
| 486186 | *CCNA_00471* |
| 486191 | *CCNA_00471* |
| 486751 | *CCNA_00471* |
