# Supplementary material for: Cell cycle constraints on capsulation and bacteriophage susceptibility
Source: eLife. 2014 Nov 25;3:e03587. doi: 10.7554/eLife.03587 (PMC4241560; doi:10.7554/eLife.03587)
Supplement: Supplementary file 3. — Strains used in this study. DOI: http://dx.doi.org/10.7554/eLife.03587.031 [file elife03587s004.docx]

**Table S3. Strains used in this study**

| ***Caulobacter crescentus*** | **Relevant characteristics** | **Reference or source** |
| --- | --- | --- |
| NA1000 | syn-1000, synchronizable variant of strain CB15 | ^60^ |
| NA1000 *ctrA401* | NA1000 derivative with the temperature-sensitive *ctrA401* allele | ^48^ |
| NA1000 *spmX-mCherry* | NA1000 derivative expressing *spmX-mCherry* at the *spmX* locus | ^75^ |
| Δ*pleC* | NA1000 derivative with in-frame deletion of *pleC* | ^80^ |
| Δ*pleC*Δ*divJ* | NA1000 derivative with in-frame deletion of *pleC* and *divJ*::Ω | ^80^ |
| Δ*mucR1*Δ*mucR2* | NA1000 derivative with in-frame deletion of *mucR1* and *mucR2* | ^12^ |
| Δ*pleC*Δ*mucR1*Δ*mucR2* | NA1000 derivative with in-frame deletion of *pleC*, *mucR1* and *mucR2* | ^12^ |
| UG1277 | Δ*mucR1*Δ*mucR2* carrying the *sciP* T24I allele | ^12^ |
| UG1278 | Δ*mucR1*Δ*mucR2* carrying the *sciP* T65A allele | ^12^ |
| UG1280 | Δ*mucR1*Δ*mucR2* carrying the *ctrA* T170A allele | ^12^ |
| *rsaA::Kan* | NA1000 derivative with pNPTS138_Δ*rsaA* integrated at the *rsaA* locus | ^68^ |
| Δ*CCNA_00162* | NA1000 derivative with in-frame deletion of *CCNA_00162* | This work |
| Δ*CCNA_00163* | NA1000 derivative with in-frame deletion of *CCNA_00163* | This work |
| Δ*CCNA_00164* | NA1000 derivative with in-frame deletion of *CCNA_00164* | This work |
| Δ*pssY* | NA1000 derivative with in-frame deletion of *pssY* | This work |
| Δ*hvyA* | NA1000 derivative with in-frame deletion of *hvyA* | This work |
| Δ*CCNA_00167* | NA1000 derivative with in-frame deletion of *CCNA_00167* | This work |
| *CCNA_00168::Tn* | NA1000 derivative with *himar1* insertion in *CCNA_00168* | This work |
| Δ*CCNA_03998* | NA1000 derivative with in-frame deletion of *CCNA_03998* | This work |
| Δ*CCNA_00470* | NA1000 derivative with in-frame deletion of *CCNA_00470* | This work |
| Δ*pleC*Δ*CCNA_00162* | NA1000 derivative with in-frame deletion of *pleC* and *CCNA_00162* | This work |
| Δ*pleC*Δ*CCNA_00163* | NA1000 derivative with in-frame deletion of *pleC* and *CCNA_00163* | This work |
| Δ*pleC*Δ*hvyA* | NA1000 derivative with in-frame deletion of *pleC* and *hvyA* | This work |
| Δ*pleC*Δ*CCNA_00167* | NA1000 derivative with in-frame deletion of *pleC* and *CCNA_00167* | This work |
| Δ*pleC* *CCNA_00168::Tn* | NA1000 derivative with in-frame deletion of *pleC* and *CCNA_00168::Tn* | This work |
| Δ*pleC*Δ*CCNA_03998* | NA1000 derivative with in-frame deletion of *pleC* and *CCNA_03998* | This work |
| Δ*pleC*Δ*CCNA_00470* | NA1000 derivative with in-frame deletion of *pleC* and *CCNA_00470* | This work |
| Δ*hvyA*Δ*CCNA_00163* | NA1000 derivative with in-frame deletion of *hvyA* and *CCNA_00163* | This work |
| Δ*hvyA*Δ*CCNA_00167* | NA1000 derivative with in-frame deletion of *hvyA* and *CCNA_00167* | This work |
| Δ*hvyA*Δ*CCNA_00470* | NA1000 derivative with in-frame deletion of *hvyA* and *CCNA_00470* | This work |
| Δ*pleC*Δ*hvyA*Δ*CCNA_00167* | NA1000 derivative with in-frame deletion of *pleC*, *hvyA* and *CCNA_00167* | This work |
| Δ*hvyA rsaA::Kan* | NA1000 derivative with in-frame deletion of *hvyA* and pNPTS138_Δ*rsaA* integrated at the *rsaA* locus | This work |
| Δ*CCNA_00163 rsaA::Kan* | NA1000 derivative with in-frame deletion of *CCNA_00163* and pNPTS138_Δ*rsaA* integrated at the *rsaA* locus | This work |
| Δ*hvyA*Δ*CCNA_00163 rsaA::Kan* | NA1000 derivative with in-frame deletion of *hvyA* and *CCNA_00163*; pNPTS138_Δ*rsaA* integrated at the *rsaA* locus | This work |
| SA1737 | NA1000 derivative with *hvyA* replaced with the *mCh-hvyA* fusion (mCherry epitope fused after residue Ala25 of HvyA) on the chromosome | This work |
| SA1951 | SA1737 with pSA480 integrated at the *hvyA* locus | This work |
| SA1984 | Δ*mucR1*Δ*mucR2* with pSA480 integrated at the *hvyA* locus | This work |
| ***Sinorhizobium fredii*** |  |  |
| *S. fredii* NGR234 | Wild-type strain | ^61^ |
| ***Sinorhizobium meliloti*** |  |  |
| Rm2011 | Wild-type strain | ^63^ |
| Rm101 | Rm2011 with Spc^R^ cassette inserted into the *Pma*CI site of *mucR* | ^62^ |
| ***Escherichia coli*** |  |  |
| EC100D | Cloning strain | Epicentre Technologies |
| S17-1 | For plasmid mobilization | ^64^ |
| XL1-Red | Mutator strain | Stratagene |
| Rosetta™(DE3)pLysS | BL21 derivative for protein expression from T7 promoter | Novagen |
