# Supplementary material for: Cell cycle constraints on capsulation and bacteriophage susceptibility
Source: eLife. 2014 Nov 25;3:e03587. doi: 10.7554/eLife.03587 (PMC4241560; doi:10.7554/eLife.03587)
Supplement: Supplementary file 4. — Plasmids used in this study. DOI: http://dx.doi.org/10.7554/eLife.03587.032 [file elife03587s005.docx]

**Table S4. Plasmids used in this study**

| **Plasmid** | **Relevant characteristics** | **Reference or source** |
| --- | --- | --- |
| pNPTS138 | Suicide vector used for gene replacement; Kana^R^ | M.R.K. Alley |
| pNPTS_Δ*00162* | pNPTS138 derivative carrying the in-frame deletion of *CCNA_00162*; Kana^R^ | This work |
| pNPTS_Δ*00163* | pNPTS138 derivative carrying the in-frame deletion of *CCNA_00163*; Kana^R^ | This work |
| pNPTS_Δ*00164* | pNPTS138 derivative carrying the in-frame deletion of *CCNA_00164*; Kana^R^ | This work |
| pNPTS_Δ*pssY* | pNPTS138 derivative carrying the in-frame deletion of *pssY*; Kana^R^ | ^36^ |
| pNPTS_Δ*hvyA* | pNPTS138 derivative carrying the in-frame deletion of *hvyA*; Kana^R^ | This work |
| pNPTS_ P*hvyA-mCh::hvyA* | pNPTS138 derivative carrying the P*hvyA-mCh::hvyA* fusion; Kana^R^ | This work |
| pNPTS_Δ*00167* | pNPTS138 derivative carrying the in-frame deletion of *CCNA_00167*; Kana^R^ | This work |
| pNPTS_Δ*00167_(_*_Δ_*_hvyA)_* | pNPTS138 derivative carrying the in-frame deletion of *CCNA_00167* for the Δh*vyA* background; Kana^R^ | This work |
| pNPTS_Δ*03998* | pNPTS138 derivative carrying the in-frame deletion of *CCNA_03998*; Kana^R^ | This work |
| pNPTS_Δ*00466* | pNPTS138 derivative carrying the in-frame deletion of *CCNA_00466*; Kana^R^ | This work |
| pNPTS_Δ*00467* | pNPTS138 derivative carrying the in-frame deletion of *CCNA_00467*; Kana^R^ | This work |
| pNPTS_Δ*00470* | pNPTS138 derivative carrying the in-frame deletion of *CCNA_00470*; Kana^R^ | This work |
| pNPTS138_Δ*rsaA* | pNPTS138 derivative for *rsaA* (*CCNA_01059*) disruption; Kana^R^ | ^68^ |
| pGS18T | For integration into *Caulobacter* chromosome; Kana^R^ | M.R.K. Alley |
| pSA480 | pGS18T derivative carrying a 719-bp fragment of *hvyA* ORF; Kana^R^ | This work |
| pJC327 | IncP broad-host range vector carrying P*_perP_*-*lacZ* translational fusion; Tet^R^ | ^40^ |
| pSA184 | pJC327 derivative carrying P*_hvyA_*-hvyA::*lacZ*; Tet^R^ | This work |
| pRKlac290 | *lacZ* transcriptional fusion vector, pRK290 derivative; Tet^R^ | ^78^ |
| pSA205 | pRKlac290 derivative carrying P*_hvyA_-lacZ*; Tet^R^ | This work |
| pSA146 | pRKlac290 derivative carrying P*_SMc00998_-lacZ*; Tet^R^ | This work |
| pHPV414 | pMini*Himar*-lacZ derivative with *oriT* | ^67^ |
| pOK12 | Cloning vector; Kana^R^ | ^71^ |
| pOK12-*hvyA* | pOK12 derivative carrying the *hvyA* ORF; Kana^R^ | This work |
| pOK12-*hvyA* _(C192S)_ | pOK12 derivative carrying the *hvyA* C192S variant; Kana^R^ | This work |
| pOK12-*hvyA* _(C192A)_ | pOK12 derivative carrying the *hvyA* C192A variant; Kana^R^ | This work |
| pOK12-*hvyA* _(H226Q)_ | pOK12 derivative carrying the *hvyA* H226Q variant; Kana^R^ | This work |
| pOK12-*hvyA* _(H226A)_ | pOK12 derivative carrying the *hvyA* H226A variant; Kana^R^ | This work |
| pOK12-*hvyA* _(D241N)_ | pOK12 derivative carrying the *hvyA* D241N variant; Kana^R^ | This work |
| pOK12-*hvyA* _(D241A)_ | pOK12 derivative carrying the *hvyA* D241A variant; Kana^R^ | This work |
| pMT375 | Low copy number plasmid for inducible expression; P*_xyl_*, Tet^R^ | ^37^ |
| pMT375-*hvyA* | pMT375 derivative carrying the *hvyA* ORF; Tet^R^ | This work |
| pMT335 | Medium copy number plasmid for inducible expression; P*_van_*, Gm^R^ | ^37^ |
| pMT335-*hvyA* | pMT335 derivative carrying the *hvyA* ORF; Gm^R^ | This work |
| pMT335-*hvyA*_(C192S)_ | pMT335 derivative carrying the *hvyA* C192S variant; Gm^R^ | This work |
| pMT335-*hvyA*_(C192A)_ | pMT335 derivative carrying the *hvyA* C192A variant; Gm^R^ | This work |
| pMT335-*hvyA*_(H226Q)_ | pMT335 derivative carrying the *hvyA* H226Q variant; Gm^R^ | This work |
| pMT335-*hvyA*_(H226A)_ | pMT335 derivative carrying the *hvyA* H226A variant; Gm^R^ | This work |
| pMT335-*hvyA*_(D241N)_ | pMT335 derivative carrying the *hvyA* D241N variant; Gm^R^ | This work |
| pMT335-*hvyA*_(D241A)_ | pMT335 derivative carrying the *hvyA* D241A variant; Gm^R^ | This work |
| pUG52 | pMT335 derivative carrying the *hvyA* ORF fused to the TAP epitope; Gm^R^ | This work |
| pSA123 | pMT335 derivative carrying the *hvyA* C192S variant fused to the TAP epitope; Gm^R^ | This work |
| pSA124 | pMT335 derivative carrying the *hvyA* H226Q variant fused to the TAP epitope; Gm^R^ | This work |
| pSA125 | pMT335 derivative carrying the *hvyA* D241N variant fused to the TAP epitope; Gm^R^ | This work |
| pSA166 | pMT335 derivative carrying the *hvyA* W178S variant fused to the TAP epitope; Gm^R^ | This work |
| pSA167 | pMT335 derivative carrying the *hvyA* D194G variant fused to the TAP epitope; Gm^R^ | This work |
| pSA168 | pMT335 derivative carrying the *hvyA* P263R variant fused to the TAP epitope; Gm^R^ | This work |
| pSA169 | pMT335 derivative carrying the *hvyA* L240R variant fused to the TAP epitope; Gm^R^ | This work |
| pSA170 | pMT335 derivative carrying the *hvyA* R161P variant fused to the TAP epitope; Gm^R^ | This work |
| pSA171 | pMT335 derivative carrying the *hvyA* H226Y variant fused to the TAP epitope; Gm^R^ | This work |
| pSA362 | pMT335 derivative carrying the *CCNA_00162* ORF; Gm^R^ | This work |
| pSA361 | pMT335 derivative carrying the *CCNA_00163* ORF; Gm^R^ | This work |
| pSA401 | pMT335 derivative carrying the *CCNA_00164* ORF; Gm^R^ | This work |
| pSA62 | pMT335 derivative carrying the *CCNA_00167* ORF; Gm^R^ | This work |
| pSA324 | pMT335 derivative carrying the *CCNA_00168* ORF; Gm^R^ | This work |
| pUG35 | pMT335 derivative carrying the *CCNA_03998* ORF; Gm^R^ | This work |
| pUG28 | pMT335 derivative carrying the *CCNA_00466* ORF; Gm^R^ | This work |
| pSA102 | pMT335 derivative carrying the *CCNA_00470* ORF; Gm^R^ | This work |
| pSA264 | pMT335 derivative carrying the *SMc00998* ORF; Gm^R^ | This work |
| pSA142 | pMT335 derivative carrying the *NGR_c12490* ORF; Gm^R^ | This work |
| pSA141 | pMT335 derivative carrying the *NGR_c19800* ORF; Gm^R^ | This work |
| pSA147 | pMT335 derivative carrying the *NGR_c36180* ORF; Gm^R^ | This work |
| pSA309 | pMT335 derivative carrying the *Atu0252* ORF; Gm^R^ | This work |
| pMT335-*mucR1* | pMT335 derivative carrying the *mucR1* ORF; Gm^R^ | ^12^ |
| pMT335-*mucR1*-long | pMT335 derivative carrying the long *mucR1* ORF (original annotation of *CC_0933*); Gm^R^ | ^12^ |
| pMT335-*mucR1* (Y97C) | pMT335 derivative carrying the *mucR1* Y97C variant; Gm^R^ | ^12^ |
| pMT335-*mucR1* (R85C) | pMT335 derivative carrying the *mucR1* R85C variant; Gm^R^ | ^12^ |
| pMT335-*Sf_a00320* | pMT335 derivative carrying the *NGR_a00320* ORF; Gm^R^ | ^12^ |
| pMT335-*Sf_c07580* | pMT335 derivative carrying the *NGR_c07580* ORF; Gm^R^ | ^12^ |
| pMT335-*At_ROS* | pMT335 derivative carrying the *Atu0916* ORF; Gm^R^ | ^12^ |
| pMT335-*Bs_MucR* | pMT335 derivative carrying the *BR0569* ORF; Gm^R^ | ^12^ |
| pMT335-*Bh_MucR* | pMT335 derivative carrying the *PRJMB_00467* ORF; Gm^R^ | This work |
| pUG48 | pMT335 derivative carrying the *sciP WT* ORF; Gm^R^ | ^12^ |
| pUG47 | pMT335 derivative carrying the *sciP* T65A variant; Gm^R^ | ^12^ |
| pET-28a | T7 expression plasmid; Kana^R^ | Novagen |
| pET-*hvyA* | pET-28a derivative expressing His_6_-HvyA_(26-272)_; Kana^R^ | This work |
| pET-*00167* | pET-28a derivative expressing His_6_-CCNA_00167_(1-208)_; Kana^R^ | This work |
| pCWR547 | pET-28a derivative expressing His_6_-SUMO-KidO | ^72^ |
| pSA354 | pCWR547 derivative expressing His_6_-SUMO-CCNA_00162_(51-422)_; Kana^R^ | This work |
| pSA352 | pCWR547 derivative expressing His_6_-SUMO-CCNA_00164_(481-620)_; Kana^R^ | This work |
| pSA342 | pCWR547 derivative expressing His_6_-SUMO-CCNA_00168_(41-198)_; Kana^R^ | This work |
| pET-47b | T7 expression plasmid; Kana^R^ | Novagen |
| pCWR496 | pET47b derivative expressing CCNA_02223_(22-289)_-His_6_; Kana^R^ | This work |
| pCWR508 | pET47b derivative expressing CCNA_00163_(101-300)_-His_6_; Kana^R^ | This work |
