# Supplementary material for: Cell cycle constraints on capsulation and bacteriophage susceptibility
Source: eLife. 2014 Nov 25;3:e03587. doi: 10.7554/eLife.03587 (PMC4241560; doi:10.7554/eLife.03587)
Supplement: Supplementary file 5. — Oligonucleotides used in this study. DOI: http://dx.doi.org/10.7554/eLife.03587.033 [file elife03587s006.docx]

**Table S5. Oligonucleotides used in this study**

| **Primer** | **Sequence 5’-3’ (Restriction sites underlined)** |
| --- | --- |
| 162_ko1 | AGA AAA AAG CTT CAG TCA TGG CTT CTA ACC GTT |
| 162_ko2 | AAA AAA GGA TCC TCG GCC TTG AGA CAA GTC GAT |
| 162_ko3 | AAA AAA GGA TCC GGC GCT TCG TTC AAG GAC AAC |
| 162_ko4 | AAA AAA GAA TTC GGC GAA CCT TAA GTC CGC CAA |
| 162_out1 | CCT GAA AGC GAA TTC GTT CAG AA |
| 162_out2 | GGC GCT CTT CTC GGA GCG CTG CTT |
| 163_ko1 | AGA AAA AAG CTT GAA GGA CTA CGA CAC CAA CGT |
| 163_ko2 | AAA AAA GGA TCC CGC GAT GTT CAG ATC AAA AGA CAA |
| 163_ko3 | AAA AAA GGA TCC GCG CAG TCT CGC TAC GGC TAC |
| 163_ko4 | AAA AAA GAA TTC GGC AAG ACT AAT GCC GGC GTC CAA |
| 163_out1 | GCG ACC CTG GTC AAG GAA TTC AA |
| 163_out2 | CCC CGC CGC GGG ATC CTG TGA AA |
| 164_ko1 | AAA AAG CTT CGG GCG TCG ATC CTG TTC TCA |
| 164_ko2 | AAA GGA TCC CGA CGA TCC GAA CGA GGA CGA |
| 164_ko3 | AAA GGA TCC GTC GCC GAG CGC ATG ACC AAG A |
| 164_ko4 | AAA GAA TTC CGG TGG ATC TAG GGC CAT CGA |
| 164_out1 | GGC CAG CAT TGG TCT CGC CGA A |
| 164_out2 | GCG GGC TTT CTG TCG TGA ACG AA |
| hvyA_ko1 | AAA GAA TTC CGA CGC AGG AAC TGT TCA T |
| hvyA_ko2 | AAA GGA TCC AAC AAT GTT TCG CGC CTT GAA |
| hvyA_ko3 | AAA GGA TCC GCC CTG TGG ACG TCG ATC GCC TA |
| hvyA_ko4 | AAA AAG CTT GTC CGT AGT TTG AGG ATC T |
| hvyA_out1 | CGCGATGAACAAGACGAACTCTAA |
| hvyA_out2 | CCGCGATCCGCTGGCGCATGAGCT |
| hvyA_up_H | AAA AAA AAG CTT CAG GTC TGC GGC GAC CTG TCG CTC AA |
| hvyA_up_B | AAA AAA GGA TCC GCC GGC CTG TGC CTG GGC GCT CGT CA |
| mCh_B | AAA AAA GGA TCC GTG TCG AAG GGT GAA GAA GAT AA |
| mCh_X | AAA TCT AGA CTT GTA GAG CTC ATC CAT GCC GCC GGT CGA |
| hvyA_down_X | AAA AAA TCT AGA GGG ATG CCG TTC ATG CCG CGC GGC CAT |
| hvyA_down_E | AAA AAA GAA TTC GCG GTG CCG GGG ACT TGA CGC TCA A |
| 167_ko1 | AAA GAA TTC GAT CGA ACC AGG TCA GGA CG |
| 167_ko2 | AAA GGA TCC CGA ACT CGC CAC GTA CGC CA |
| 167_ko3 | AAA GGA TCC GCT TAG AGT TCG TCT TGT TCA TC |
| 167_ko4 | AAA AAG CTT GGG TCG AAG GCT TCA GCT CG |
| 167_out1 | GAT CAG TTG GCG ACG CTT CTC |
| 167_out2 | CTG AGC CAT CTC GAA CCA GAC |
| 167_ko5 | AAA GAA TTC TCC CGG CAA TGC ATG GTC CG |
| 167_ko6 | AAA AGA TCT CGA ACT CGC CAC GTA CGC CA |
| 167_out3 | TATTCAGGGATTCGCGCGCTG |
| 3998_ko1 | AAA AAA GAA TTC ACC TCT ACC GTC TGC TGA GCC TGA A |
| 3998_ko2 | AAA AAA GGA TCC ATA GTT CAA CCC CAC AAT CAG AA |
| 3998_ko3 | AAA AAA GGA TCC ATT GAT GGC TTG GAG GCC GAG CTG AA |
| 3998_ko4 | AAA AAA AAG CTT ATC GCG TCG GTG TCG TCC CTC AAA |
| 3998_out1 | CGT GCC GGG GGG CGC AGG CGA A |
| 3998_out2 | CGC TCA TGG CCA CTC CAG CCG CCT AA |
| 466_ko1 | AAA AAA CAA TTG ATG AAT TCC AGG CTA TGC CCA A |
| 466_ko2 | AAA AAA GGA TCC CAT TGG GAA ATA GTC TTT ACA ATA AA |
| 466_ko3 | AAA AAA GGA TCC TAC AAC TGT CTA CAG TTG TTG AAA |
| 466_ko4 | AAA AAA AAG CTT CCG GAT GCG ATC ACC GCC ATT AAA |
| 466_out1 | CGG GGG TCC TTC TAC ATC GCT A |
| 466_out2 | GTC GAG GGC AAA GCG CCA CAA |
| 467_ko1 | AAA AAG CTT GCG ATG GAA GCA AGA GCC AG |
| 467_ko2 | AAA GGA TCC GTT GGC TCG AAT GGA CTT CAT A |
| 467_ko3 | AAA GGA TCC GCC TAG GCT GCC GCA GAT C |
| 467_ko4 | AAA GAA TTC TGT CCA AGG CTG CCT TGG AG |
| 467_out1 | CCA ACA CCG TCA AGA CCG TCC AA |
| 467_out2 | AGC ATA GTT CGC GTC AGA GGG AA |
| 470_ko1 | AAA GAA TTC CAA GGT GCT GTT CCT AGG CTC |
| 470_ko2 | AAA GGA TCC CCC GCG AGC TTC CAT CTA GG |
| 470_ko3 | AAA GGA TCC GCA TCC CGC CGA CAA GG |
| 470_ko4 | AAA AAG CTT GGC CTT GGG CAG ATC TAC TC |
| 470_out1 | GCA TCC TGG CCA ACG ACA CC |
| 470_out2 | GTC TGC TCG CTA CGA CGT GG |
| PhvyA_B | AAA AGA TCT GCT GGC TTA GCG CGA TGA |
| PhvyA_P | AAA CTG CAG TTT CGC GCC TTG AAC ATG CT |
| Sm998_B | AAA GGA TCC CTG TGA CGA GCA CAG GGA T |
| Sm998_P | AAA CTG CAG GTG ATG ATT GTG CGC ATG TCT GT |
| hvyA_N | AAA AAA CAT ATG TTC AAG GCG CGA AAC ATT GTT |
| hvyA_E | AAA GAA TTC AAC GGA CCC TTG CGC GAG AAA CGT TA |
| hvyA_CTIF | AAA AAA GAA TTC GGC GAT CGA CGT CCA CAG GGC GGT |
| hvyA_in_B | AAA AGG ATC CGG TTT CGC CGA CCT TTG CCG A |
| 162_N | AAA AAA CAT ATG CGA ATT CTA ACG TGT TCG GCC AT |
| 162_M | AAA AAA CAA TTG TCA GAA CTG CAG GGC GAC CGA A |
| 163_N | AAA AAA CAT ATG GAC GGC TCC AGG TTT GAA ACT TCG AA |
| 163_E | AAA AAA GAA TCC TAG TCC GCA TAG TAC TTT CGA TAA |
| 164_N | AAA AAA CAT ATG CGG ACT AGC AAG CGG TT |
| 164_E | AAA AAA GAA TTC TAC TGA TCG GCT GTC TTG GTC AT |
| 167_N | AAA AAA CAT ATG GCG TAC GTG GCG AGT TCG ATG TT |
| 167_E | AAA AAA GAA TTC TAA GCT GCG CGC GGT TTA CTA AA |
| 168_N | AAA AAA CAT ATG AGA CGC TTT TCC ATG ATC GGT TT |
| 168_E | AAA AAA GAA TTC TAG AAG AAG CGC TCA CCA ATG CGG A |
| 3998_N | AAA AAA CAT ATG CCA GAC AAA CGT ATA AAG CTT |
| 3998_E | AAA AAA GAA TTC TAG GCG CAC ACG CGC TTC A |
| 466_N | AAA AAA CAT ATG CGC ATC GCA GTT TAT TGT AAA |
| 466_M | AAA AAA CAA TTG TTA TTG CCG TTT CAA CAA CTG TAG ACA |
| 470_N | AAA CAT ATG GAA GCT CGC GGG TCA GG |
| 470_E | AAA GAA TTC TTA CGT GGC GAC GTT CGG CA |
| Sm998_N | AAA AAA CAT ATG CGC ACA ATC ATC ACC ATG GCG A |
| Sm998_E | AAA AGA ATT CAT TGT GCG CTG ACG CTG CCG ACG GCG AGA |
| Sf12490_N | AAA AAA CAT ATG CAC AAG ACA ATC GCC AAG A |
| Sf12490_M | AAA AAA CAA TTG TTA CTC GGT GCG GAC CGT ACC GA |
| Sf19800_N | AAA AAA CAT ATG AAA CGC TTG ATC GCG CAT GTC T |
| Sf19800_E | AAA AAA GAA TTC AGA TCG CCT CCG CTC CGC CGA |
| Sf36180_N | AAA AAA CAT ATG GCA TTT TGG ACC GGG GTA AA |
| Sf36180_E | AAA AAA GAA TTC ACT TGC CGA CGG AGC CGA CCA |
| At252_N | AAA AAA CAT ATG ATG AAC GCA CCG CTG GCG CGT GCT CT |
| At252_E | AAA AAA GAA TTC AGC GCA ACG CGC CGA CCA GAA |
| hvyA_C192S | CAA GCT CTA TGG CGA CAG CGA GGA CTA CGT CC |
| hvyA_C192S_as | GGA CGT AGT CCT CGC TGT CGC CAT AGA GCT TG |
| hvyA_C192A | AAG CTC TAT GGC GAC GCC GAG GAC TAC GTC CT |
| hvyA_C192A_as | AGG ACG TAG TCC TCG GCG TCG CCA TAG AGC TT |
| hvyA_H226Q | GCG GGG CGA GGG GCA AGC GGT CCT GAT GGT G |
| hvyA_H226Q_as | CAC CAT CAG GAC CGC TTG CCC CTC GCC CCG C |
| hvyA_H226A | GCG CGG GGC GAG GGG GCC GCG GTC CTG ATG GT |
| hvyA_H226A_as | ACC ATC AGG ACC GCG GCC CCC TCG CCC CGC GC |
| hvyA_D241N | GGC GAC TGG GTG CTC AAC AAC CTC ACG CCG TG |
| hvyA_D241N_as | CAC GGC GTG AGG TTG TTG AGC ACC CAG TCG CC |
| hvyA_D241A | GCG ACT GGG TGC TCG CCA ACC TCA CGC CGT GG |
| hvyA_D241A_as | CCA CGG CGT GAG GTT GGC GAG CAC CCA GTC GC |
| 162_in_N | AAA AAA CAT ATG CAA AAG GCT GGC GGT TTC ACC GTC T |
| 162_in_S | AAA AAA GAG CTC AGA ACT GCA GGG CGA CCG AAG CCA CCA |
| 163_in_N | AAA AAA CAT ATG CGC TCG CTC GCC GCG CGC GT |
| 163_His_E | AAA GAA TTC AGT GGT GGT GGT GGT GGT GCA GGG ACT CGC CAA |
| 164_in_N | AAA AAA CAT ATG GCA CGT TTC GGG GGA GAC CTG A |
| 164_in_S | AAA AAA GAG CTC ACG GGT CCT GAA TGC TGT TGG CCA GA |
| hvyA_short | AAA CAT ATG GGC GGG ATG CCG TTC ATG C |
| 167_in_E | AAA GAA TTC TAG AAA TCG TTG CGG ATC CAA AGC AGG T |
| 168_short | AAA AAA CAT ATG GCG GGA GCG GCG CAA GCG GTC GA |
| bla_N | AAA ACA TAT GGA CGA CAT GCC GGC CAA CT |
| bla_His_E | AAA GAA TTC AGT GGT GGT GGT GGT GGT GTC GCT TGG GCC CCG |
